# The role of motivation and cultural dialects in the in-group advantage for emotional vocalizations

**DOI:** 10.3389/fpsyg.2013.00814

**Published:** 2013-10-30

**Authors:** Disa A. Sauter

**Affiliations:** Department of Social Psychology, University of AmsterdamAmsterdam, Netherlands

**Keywords:** emotion, in-group advantage, vocalizations, non-verbal communication, motivation

## Abstract

It is well-established that non-verbal emotional communication via both facial and vocal information is more accurate when expresser and perceiver are from the same cultural group. Two accounts have been put forward to explain this finding: According to the dialect theory, culture-specific learning modulates the largely cross-culturally consistent expressions of emotions. Consequently, within-group signaling benefits from a better match of the “emotion dialect” of the expresser and perceiver. However, it has been proposed that the in-group advantage in emotion recognition could instead arise from motivational differences in the perceiver, with perceivers being more motivated when decoding signals from members of their own group. Two experiments addressed predictions from these accounts. Experiment 1 tested whether perceivers' ability to accurately judge the origin of emotional signals predicts the in-group advantage. For perceived group membership to affect the perceivers' motivation, they must be able to detect whether the signal is coming from an in-group or out-group member. Although an in-group advantage was found for in-group compared to out-group vocalizations, listeners were unable to reliably infer the group membership of the vocalizer. This result indicates that improved recognition of in-group signals can occur also when the perceiver is unable to judge whether signals were produced by in- or out-group members. Experiment 2 examined the effects of expected and actual group membership of signals on emotion recognition by manipulating both orthogonally. The actual origin of the stimulus was found to significantly affect emotion recognition, but the believed origin of the stimulus did not. Together these results support the notion that the in-group advantage is caused by culture-specific modulations of non-verbal expressions of emotions, rather than motivational factors.

## Introduction

### The in-group advantage

Emotional signals are largely shared across cultural groups. However, a consistent finding in cross-cultural research on non-verbal emotional communication is that recognition accuracy is higher when expresser and perceiver are from the same cultural group (see Elfenbein and Ambady, 2002a for a meta-analysis). This pattern of in-group advantage has been found for visual cues in the form of both facial expressions (e.g., Ekman et al., 1969; Haidt and Keltner, 1999) and postural cues (Tracy and Robins, 2008). The in-group advantage has also been found for auditory signals, specifically speech prosody (Scherer et al., 2001) and non-verbal vocalizations (Sauter et al., 2010b). Two mechanisms have been proposed to explain the in-group advantage: Perceivers may be more motivated when judging in-group signals, or the physical expressions may be modulated by cultural learning, which can lead to a disadvantage when encoder and perceiver are from different cultures.

### The motivation account

The in-group advantage in emotion recognition could arise from motivational differences in the perceiver when judging in- vs. out-group expressions (e.g., Thibault et al., 2006). According to this view, the extent to which perceivers are motivated to attempt to take the expresser's perspective, and thus decode their emotional state, depends on the extent to which they identify with the expresser. This builds on the findings that others who are perceived to be in-group members are attended more to, and are also typically evaluated more positively than out- group members (see Tajfel and Billic, 1974). In the context of emotion communication, Thibault et al. (2006) suggest that observers may engage in more challenging strategies when decoding in-group expressions. According to this view, ethnic cultural groups constitute a subset of social groups, which depend on group identification. An in-group advantage would thus be expected for emotional communication between social groups of any kind, including groups differentiated by culture and/or ethnicity.

Thibault et al. provided empirical support for the motivational account in a study where basketball players and non-basketball players judged the emotional facial expressions of individuals who they were told were either basketball players or non-basketball players (Thibault et al., 2006). Participants who themselves played basketball were expected to consider other basketball players as their in-group, and hence be more accurate in their judgments of their emotional expressions. The authors found that recognition accuracy was affected by the group membership of the judge as well as the perceived group membership of the target, and concluded that “group identification influences decoding accuracy” (p. 682).

A study by Young and Hugenberg found further support for a motivational account of the in-group advantage for facial expressions (2010). They elicited an in-group advantage while holding the culture of the expresser and perceiver constant, by creating a minimal-group paradigm using fake feedback from a personality test. They found that in-group faces were processed more configurally than out-group faces. Given that configural processing is beneficial for the decoding of emotional facial expressions (Calder et al., 2000), the authors suggest that this processing bias may underlie the advantage for in-group judgments of facial expressions. They further argue that the processing bias is motivationally driven, based on the fact that the in-group advantage as well as configural processing difference disappears under reduced exposure time. To what extent this mechanism would apply to emotional signals other than facial expressions is unclear, given that this kind of configural processing has primarily been studied with faces.

Two studies of facial mimicry have also found support for a motivational account. In a related study to that by Thibault et al. (2006), using the same participants and stimuli, Bourgeois and Hess (2008; Experiment 2) found that perceivers displayed more mimicry for in-group as compared to out-group displays of sadness, although no effect was found for displays of anger or happiness. The authors concluded that the level of facial mimicry varies as a function of group membership, at least for some emotional states. Similarly, van der Schalk et al. (2011) examined facial mimicry to in-group and out-group expressions of emotions, presenting expressers either as a student of psychology (in-group) or as a student of economics (out-group). In that study, mimicry of anger and fear facial expressions, but not happiness, was found to be affected by group membership, with more mimicry occurring to in-group displays.

Together, these studies suggest that, at least for the perception of emotional facial expressions, signals may be affected by the extent to which perceivers identify with the expresser and judge them to belong to their own group.

### The dialect account

An alternative explanation of the in-group advantage is that culture-specific learning modulates non-verbal expressions of emotions. This is the account advanced by proponents of the dialect theory (Elfenbein and Ambady, 2002a). According to this view, within-group signaling benefits from a better match of the “emotion dialect” of the expresser and perceiver and hence results in improved accuracy.

One study that tested this account directly was conducted by Elfenbein et al. (2007). They asked individuals from Canada and Gabon to try to communicate a range of emotions to a friend using facial expressions. Analyzing the facial expressions using the Facial Action Coding System (FACS), they found that the muscle movements in the two groups largely converged on the expressions posited to be universal prototypes (Ekman and Friesen, 1978). In addition, and in support of the dialect account, reliable cultural differences emerged, which went beyond idiosyncratic differences of individual posers. The same expressions were then used in an emotion recognition task with participants from Canada and Gabon. Greater accuracy was found for judgments of in-group expressions, and although this pattern was consistent across emotions, a larger in-group advantage was found for those emotional states that exhibited greater differences in muscle movements. In fact, no in-group advantage was found when stimulus materials from the different groups were constrained to have an identical appearance (see Elfenbein and Ambady, 2002b; Matsumoto, 2002 for a discussion of this issue). The authors concluded that cross-cultural differences in expressive style underlie the in-group advantage for emotional expressions, rather than motivational or other factors.

### The current study

Two experiments are presented which were designed to test predictions derived from the motivational (Experiments 1 and 2) and dialect (Experiment 2) accounts. Experiment 1 sought to examine the relationship between the in-group advantage and perceivers' ability to judge whether an emotional expression was produced by an in- or out-group member. For group-based motivational mechanisms to work, perceivers must be able to reliably judge whether a signal was produced by an in- or out-group member. Experiment 1 also examined whether individual differences in the ability to identify the group membership of emotional expressions would predict the extent to which perceivers show an in-group advantage. Experiment 2 aimed to test the relative contributions of motivation and cultural dialect to the in-group advantage for emotional vocalizations in a design that orthogonally manipulated the believed and actual cultural origin of the stimuli.

Research investigating the underlying mechanisms of this phenomenon has focused on facial expressions, although the in-group advantage has been found for facial, postural, and vocal signals (Elfenbein and Ambady, 2002a). There has been a call for the inclusion of non-verbal channels of communication other than the face, such as the voice (Elfenbein et al., 2007). A number of studies have found evidence for an in-group advantage also in emotional speech prosody (Scherer et al., 2001; Thompson and Balkwill, 2006; Pell et al., 2009).

The stimuli employed in these experiments were non-verbal vocalizations of emotions, such as cheers, laughs, and sighs. Group differences based on ethnicity and language are often obvious from facial or speech features, but non-verbal vocalizations offer a type of signal from which group membership may not be easily inferred. Both experiments used ethnic cultural groups to define in- and out-groups, since this is the level at which cultural dialects would be likely to work most extensively.

## Experiment 1

Experiment 1 firstly sought to replicate previous findings of an in-group advantage for non-verbal vocalizations of emotions (Sauter and Scott, 2007; Sauter et al., 2010b). It was expected that degree of in- or out-group membership would vary with cultural distance. The perceivers consisted of a group of Dutch listeners, and so Dutch vocalizations were in-group stimuli. A British set of sounds were close out-group stimuli, and a set of Namibian vocalizations were distant out-group stimuli. The experiment then tested two predictions derived from the motivational account. The first hypothesis was that perceivers should be able to reliably judge whether non-verbal vocalizations of emotions were produced by in- or out-group members, assuming that an in-group advantage was found. The second hypothesis was that individuals who were better at judging the group membership of the vocalizations would show a larger in-group advantage.

### Methods

#### Stimuli

The stimuli were taken from sets of non-verbal vocalizations of positive emotions (Dutch: Sauter et al., 2010a British: Sauter and Scott, 2007; Namibian: Sauter et al., 2010b). Each stimulus set consisted of six vocalizations per emotion, for the four emotions triumph, relief, amusement and sensual pleasure, resulting in 24 sounds per group and a total of 72 stimuli. Stimulus sex was balanced within each condition, with equal numbers of male and female tokens of each emotion for Dutch, British, and Namibian sounds, respectively. No exact age range was specified for the individuals producing vocalizations (given that the Namibian sample do not count age), but children, adolescents, and elderly adults were not included. The entire stimulus set was normalized for peak amplitude and was digitized at 41 kHz.

#### Participants

Thirty students (14 males; mean age 20.42 years, range 19–25 years) from the University of Amsterdam participated in the experiment in exchange for research credits. All participants were Dutch and reported having normal hearing.

#### Design and procedure

Participants were tested individually and completed two task blocks in one session. The first task tested emotion recognition, and the second block examined judgments of group origin of the same stimuli. Participants were informed that the experiment consisted of two parts and that they would receive the instructions for the second part after completing the first part. This was to ensure that participants would complete the emotion recognition task without knowing that the sounds were produced by individuals from different cultural groups. Thus, any in-group advantage would be independent of listeners' conscious awareness that the stimuli they heard originated from several different cultural groups. Sounds were delivered via headphones using the Psychophysics toolbox (Brainard, 1997) for MATLAB (Mathworks Inc., Natick, MA) running on a MacBook Pro laptop. Before each block, participants were given written instructions and had the opportunity to ask questions. After completing both tasks, participants were debriefed about the purpose of the study. The project was approved by the University of Amsterdam Department of Psychology ethics committee, and informed consent was obtained from all participants.

#### Emotion recognition task

The emotion recognition block consisted of a forced-choice categorization task with four response alternatives: triumph (in Dutch: *success)*, relief (in Dutch: *opluchting)*, amusement (in Dutch: *vermaak)*, and sensual pleasure (in Dutch: *genot)*. The written instructions for the emotion recognition task included a scenario for each of the four emotions (see Sauter et al., 2010a). The stimuli consisted of six tokens of each of these emotions from each of the three cultural groups (Dutch, British, and Namibian), resulting in a total of 72 sounds. The stimuli were played in a random order, and participants responded using key presses to select between response alternatives displayed on the screen in Dutch alphabetical order.

#### Group identification task

The group identification task consisted of a forced-choice task in which participants were asked to judge where the person producing each stimulus was from. The three response options were “the Netherlands,” “a different country in Europe,” and “a different country outside Europe.” The same stimuli as in the emotion recognition task were used. Again, the stimuli were played in a random order, and participants responded using key presses. Response alternatives were displayed in order of proximity from the Netherlands.

### Results

In forced-choice tasks with multiple response alternatives, performance for a particular category can be artificially inflated by the disproportionate use of that response. Unbiased hit rates (Hu scores, see Wagner, 1993) were calculated to control for this bias in both tasks. Hu scores are calculated separately for each participant for each condition (see Table A1 for results for individual emotions), and a score of one denotes perfect performance and a score of zero denotes no correct responses. As Hu scores are proportional, they were arcsine transformed before use in statistical tests.

#### Is there an in-group advantage in emotion recognition from vocalizations?

**Figure 1:**
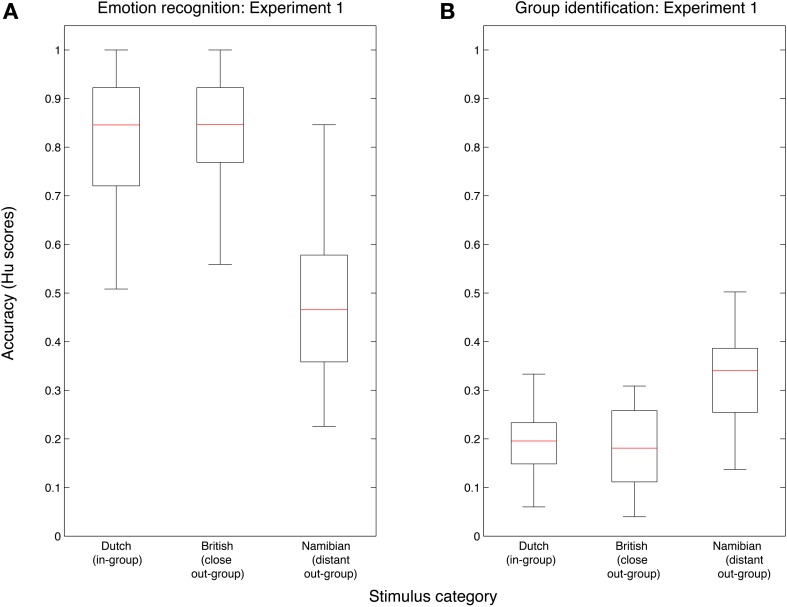
**Performance on the emotion recognition task (A) and the group identification task (B) in Experiment 1**. Data are plotted by stimulus group using (raw) Hu scores, with Dutch, British, and Namibian stimuli shown separately. Red lines are the medians, box edges are the 25th and 75th percentiles, and the whiskers extend to the most extreme data points excluding outliers.

In order to confirm whether listeners performed better with in-group as compared to out-group vocalizations on the emotion recognition task, the Hu scores from block 1 were compared using paired-sample *t*-tests, with separate tests to contrast performance with Dutch vocalizations to that with British and Namibian sounds, respectively. Dutch sounds were significantly better recognized than Namibian sounds [*t*_(29)_ = 9.61, *p* < 0.001], see Figure 1A. However, no difference was found in recognition levels between Dutch and British sounds [*t*_(29)_ = 0.82, *p* = 0.42], see Figure 1A. These results indicate that an in-group advantage is present in the recognition of emotional vocalizations from distantly-related, but not very closely-related, cultural groups.

#### Can listeners identify group membership from sounds?

To test whether participants were able to identify group membership at better-than-chance levels, Hu-scores from block 2 were compared to chance scores in paired-sample *t*-tests, separately for the Dutch, British, and Namibian vocalizations. Performance was not better than chance for any set of vocalizations (see Figure 1B). In fact, performance was significantly worse than chance for both Dutch [*t*_(29)_ = −10.78, *p* < 0.001] and British [*t*_(29)_ = −10.44, *p* < 0.001] vocalizations, likely due to the particularly high rate of confusions between these two groups. Performance was no different than chance for Namibian sounds [*t*_(29)_ = −0.52, *p* = 0.61]. These results show that listeners are not able, on a group level, to reliably judge group membership from emotional vocalizations.

#### Does group identification predict the in-group advantage?

**Figure 2:**
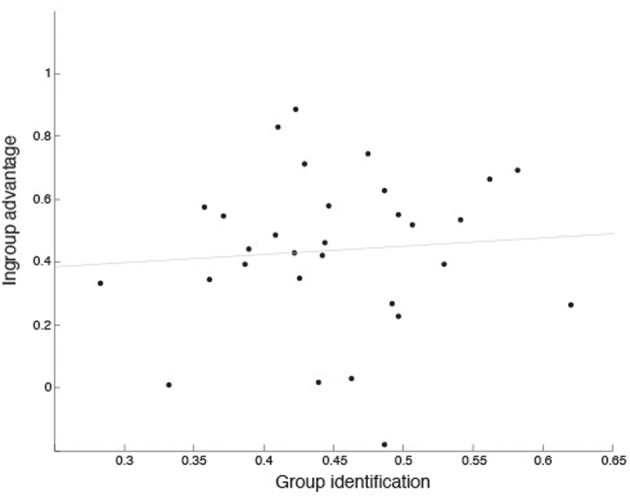
**Relationship between in-group advantage and performance on group identification in Experiment 1**. In-group advantage was calculated as accuracy for Dutch (in-group) minus Namibian (out-group) stimuli, using Hu scores. Group identification scores are mean accuracy, calculated using Hu scores, across all three stimulus groups.

To examine whether individual listeners' ability to identify the expresser's group membership would predict the in-group advantage they displayed, a linear regression was performed. The independent measure was performance on the group identification task, using Hu-scores. The dependent measure was the in-group advantage, calculated as performance on the emotion recognition task for Dutch as compared to Namibian stimuli, given that no significant difference was found for performance with Dutch as compared to British stimuli. The regression analysis was not statistically significant (*r*^2^ = 0.01, *p* = 0.68; see Figure 2), indicating that the ability to judge whether sounds were produced by in- or out-group individuals did not predict the advantage displayed for recognizing in-group vocalizations.

### Discussion

The results of this experiment showed that perceivers were not able to reliably judge whether non-verbal vocalizations of emotions were produced by in- or out-group members. Other non-verbal signals of emotions, such as facial expressions, typically allow perceivers to infer group membership even of visually similar cultural groups, such as Australians and Americans (Marsh et al., 2007), and Japanese and Japanese-Americans (Marsh et al., 2003, but see also Matsumoto, 2007). Perceivers are also able to infer group identity from speech segments, as shown in a study by Walton and Orlikoff (1994). Using sustained vowel sounds, they found that listeners could identify the speakers' race correctly 60% of the time in a two-way forced choice task. The contrast between the current study and studies of facial expressions and speech may suggest that non-verbal vocalizations are an unusual class of communicative signal in that they do not carry information about group identity.

The fact that an in-group advantage was found in the current study even though listeners were unable to tell whether sounds were produced by in- or out-group members demonstrates that motivational mechanisms are not necessary for an in-group advantage to occur. This does not rule out the possibility that motivational mechanisms contribute to the in-group advantage in cases where perceivers can accurately judge, or are explicitly told about, the group membership of the encoder. However, the current results also found no relationship between individuals' ability to judge group membership and the size of the in-group advantage that they displayed, further supporting the notion that the in-group advantage for non-verbal vocalizations of emotions does not rely on motivational factors in perceivers.

Consistent with previous research and similar to other types of non-verbal signals (Elfenbein and Ambady, 2002a; Sauter et al., 2010b), the current results show that there is an in-group advantage for non-verbal vocalizations of emotions. However, the current study found no in-group advantage for Dutch as compared to British stimuli. This is in somewhat inconsistent with a previous study in which British listeners performed better than Swedish listeners with British stimuli (Sauter and Scott, 2007). The difference between the two sets of results may be partly due to the fact that the current experiment employed a within-subject design. The current study also had fewer response alternatives as it left out the least well recognized stimulus type used by Sauter and Scott, resulting in near-ceiling accuracy for both Dutch and British sounds. Another possibility is that Swedish vocalizations are more similar to British ones than are Dutch ones. Further studies are needed to examine similarities between the vocalizations of difference cultural groups in terms of physical cues, and the relationship of these to listeners' perception (see also Pell et al., 2009 for a discussion of cultural out-group distance in the context of speech prosody).

## Experiment 2

Experiment 1 showed that listeners cannot reliably judge whether non-verbal vocalizations of emotions were produced by members of their own cultural group or not. In Experiment 2, participants' belief about the origins of the sounds was manipulated in order to allow for a test of the relative contributions of motivation and cultural dialects to the in-group advantage. In a 2 × 2 design, Dutch participants heard emotional vocalizations in two blocks, each of which they were told contained either Dutch or foreign stimuli. In each block, the actual cultural origin of the stimuli was mixed so that an equal number of Dutch and foreign stimuli were presented. According to the dialect account, an in-group advantage should be found based on the actual origin of the stimuli, while according to the motivation account an advantage should be found for the recognition of stimuli that the participants believed were produced by in-group members.

### Methods

#### Stimuli

The stimuli were taken from sets of non-verbal vocalizations of positive emotions, but using only Dutch and Namibian sounds (Dutch: Sauter et al., 2010a; Namibian: Sauter et al., 2010b). Each stimulus set consisted of six vocalizations per emotion, for the eight emotions anger, fear, triumph, relief, amusement, surprise, sadness, and sensual pleasure, resulting in 48 sounds per group and a total of 96 stimuli. The British stimuli were excluded in Experiment 2, because no in-group advantage was found for Dutch as compared to British stimuli in Experiment 1. In addition, the set of the emotions was expanded from that used in Experiment 1. This was to get a more nuanced measure of emotion recognition which could also detect small effects. Stimulus sex was balanced within each condition, with equal numbers of male and female tokens of each emotion for Dutch, British, and Namibian sounds, respectively. The entire stimulus set was normalized for peak amplitude and was digitized at 41 kHz.

#### Participants

Thirty students participated in the experiment. The participants received no reward for taking part. All participants were Dutch and reported having normal hearing. One participant was excluded because she did not have Dutch parents, resulting in a final sample of 29 participants (15 males; mean age 20.39 years, range 18–24 years).

#### Design and procedure

Participants were tested individually and completed two task blocks in one session. In one block participants were informed that they would hear vocalizations produced by Dutch individuals, and in the other block they were told that they would hear sounds by foreign individuals. Block order was counter-balanced across participants. In actual fact, within each block, half of the stimuli were Dutch and the other half of the stimuli were Namibian, with each set balanced for stimulus emotion and sex. Before each block, participants were given written instructions and had the opportunity to ask questions. The written instructions for the emotion recognition task included a scenario for each of the emotions (see Sauter et al., 2010a). In both blocks participants performed a forced-choice categorization task with eight response alternatives: triumph (in Dutch: *success)*, anger (in Dutch: *woede*), relief (in Dutch: *opluchting)*, fear (in Dutch: *angst*), amusement (in Dutch: *vermaak)*, sadness (in Dutch: verdriet), sensual pleasure (in Dutch: *genot)*, and disgust (in Dutch: *walging*). The stimuli were played in a random order, and participants responded using key presses, with response alternatives displayed on the screen in Dutch alphabetical order. Sounds were delivered via headphones using the Psychophysics toolbox (Brainard, 1997) for MATLAB (Mathworks Inc., Natick, MA) running on a MacBook laptop. After completing both tasks, participants were asked what they thought the purpose of the study was, in order to examine whether they were aware that the instructions they had received were untrue. None of the participants had seen through the deception. The project was approved by the University of Amsterdam Department of Psychology ethics committee, and informed consent was obtained from all participants.

### Results

Hu scores were calculated to yield an accuracy measure controlling for any response biases, and scores were arcsine transformed before use in statistical tests (see Wagner, 1993).

#### What are the effects of expected and actual group belonging on emotion recognition?

**Figure 3:**
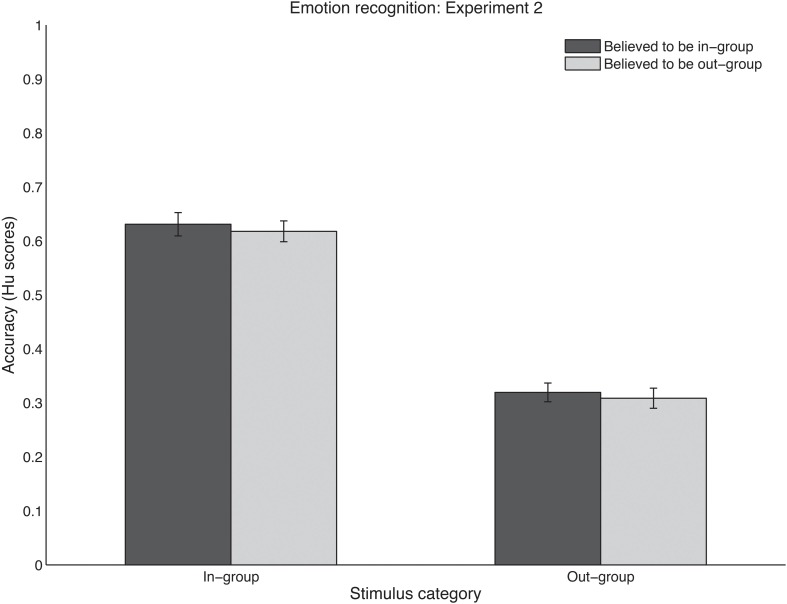
**Recognition performance in Experiment 2 using (raw) Hu scores, for in-group stimuli (left cluster) and out-group stimuli (right cluster). Sounds were believed to be in-group (dark bars) or out-group (light bars)**. Scores range between zero (no accurate responses) and one (perfect performance). Error bars denote one standard error of the mean.

How listeners' performance was affected by the expected and actual group belonging of the stimuli was tested in an ANOVA with the two within-subjects factors expected stimulus group (Dutch vs. foreign) and actual stimulus group (Dutch vs. Namibian). A significant main effect was found for actual stimulus group [*F*_(1, 28)_ = 281.20, *p* < 0.0001], with Dutch stimuli being recognized more accurately than Namibian sounds (see Figure 3). No main effect was found for expected stimulus group [*F*_(1, 28)_ = 0.39, *p* = 0.54] and no interaction was found between the two factors [*F*_(28, 115)_ = 0.01, *p* = 0.92].

### Discussion

Experiment 2 shows that, for non-verbal vocalizations of emotion, in-group sounds are more accurately understood, regardless of what listeners believe the cultural origin of the sounds to be. This pattern of results lends support to the dialect account (Elfenbein and Ambady, 2002a), in that it demonstrates that differences in the actual cultural origin of emotional signals produce differences in recognition rates. This in line with the idea of small physical differences in the affective signals of different groups, which are transmitted via cultural learning. For facial expressions, such differences could involve variations in the muscles used to signal particular states, as well as the intensity and dynamics in the activation of those muscles. These are likely acquired via implicit norms surrounding the regulation and display of emotions, including display rules (see Mesquita and Frijda, 1992, for a discussion). For vocalizations, differences may be expressed in amplitude, spectral, or pitch cues, and for vocal signals the native language of the producer may additionally influence the phonetic properties of the expressions (see Pell et al., 2009).

The results of this study fail to support a motivational explanation for the in-group advantage of emotional vocalizations. However, motivation has previously been found to have an effect in designs where stimuli were equivalent (Thibault et al., 2006; Young and Hugenberg, 2010). One difference between the current and previous studies may be the use of ethnic cultural groups in the current experiments, as opposed to cultural groups based on other aspects of identity (playing basketball or results on a personality test). Another difference is the use of vocal stimuli in the current study, in contrast to facial displays in previous research. Group membership, at least with regards to ethnicity, may be a less salient cue in vocalizations than in other non-verbal displays, such as facial expressions. From faces, perceivers are able to make reliable group judgments even for individuals who are visually similar (Marsh et al., 2003, 2007). Furthermore, in the current experiment, group membership was not emphasized to participants, and it cannot be ruled out that believed group membership may have affected recognition accuracy if it had been made more salient and/or relevant to participants. Nevertheless, the results of this study show that actual, but not believed, group belonging of non-verbal emotional vocalizations, underlie the in-group advantage in a cross-cultural paradigm.

## General discussion

The current set of experiments set out to test predictions derived from two accounts of the in-group advantage for emotional signals. Experiment 1 tested predictions made on the basis of the motivational account, and Experiment 2 tested predictions of both the motivational and dialect theories. Experiment 1 failed to find any relationship between the in-group advantage and perceivers' ability to judge whether an emotional expression was produced by an in- or out-group member, as measured by individual differences. Furthermore, an in-group advantage was found despite listeners being unable to accurately judge whether stimuli were produced by in- or out-group members. This indicates that motivational factors is not necessary for an in-group advantage to emerge for emotional stimuli, given that perceivers must be able to infer whether a signal was produced by an in-group or out-group member in order for motivational mechanisms to work.

In Experiment 2, the believed and actual cultural origin of the stimuli were orthogonally manipulated in order to examine the relative contributions of motivation and cultural dialect to the in-group advantage. As would be predicted by the dialect account, a strong effect was found for the actual group membership of the stimuli. The dialect account is consistent with co-evolutionary accounts of signal production and perception. According to this view, mechanisms of production and perception have evolved under reciprocal pressure toward a shared set of communication features (Gentner and Margoliash, 2003; see also Smith et al., 2005 for supporting evidence for emotional signals).

The believed group of the stimuli did not affect recognition rates, in contrast to the prediction derived from the motivational account. Thus, across two experiments, the current study failed to find support for a motivational mechanism underlying the in-group advantage for emotional communication. It is worth noting that the current study did not employ a balanced design, which is the most robust way for studying the in-group advantage (Matsumoto, 2002). The development of new, validated corpora from several cultural groups at varying distance from each other would facilitate the use of balanced designs. This is particularly important given that an in-group advantage in uni-directional designs may occur due to a difference in decoding-ease of the stimuli (Matsumoto, 2002). This is, however, unlikely to be the case for the stimuli used in the current study, as a bi-directional in-group advantage has already been demonstrated for a Namibian—British comparison of those sounds (Sauter et al., 2010b). The fact that Namibian listeners recognized Namibian stimuli better than British sounds suggests that, rather than the Namibian stimuli being of inferior quality to the Dutch and British ones, how easily the sounds are decoded depends on the cultural background of the listener.

The findings from the current study raise some questions about the constraints under which a motivational mechanism may apply. One possibility is that motivation may primarily affect perception in the context of groups that do not differ in their physical signals. Previous studies that have found support for a motivation explanation have contrasted students from different disciplines (van der Schalk et al., 2011), basketball players with non-basketball players (Thibault et al., 2006), or people with different personality test scores (Young and Hugenberg, 2010). In cross-cultural contexts, the role of motivation may thus play a less pronounced role. However, whether motivational mechanisms operate as an out-group bias, rather than eliciting an in-group advantage has recently been questioned (Elfenbein, 2013). An out-group bias could be expected to occur in cross-cultural settings with foreign groups that are perceived, for example, as of low status.

The current results also indicate that emotion recognition may work differently to emotional mimicry. Two studies to date have found more mimicry for believed in-group as compared to believed out-group displays of emotions, while controlling for the physical features of the stimuli (Bourgeois and Hess, 2008; van der Schalk et al., 2011). It is worth noting that the current study used auditory stimuli, while most previous studies have used facial expressions of emotions. However, van der Schalk et al. also note that the theoretical implications of their results are not clear, given the small effect sizes and the inconsistency of the effect across emotions.

One way to further explore predictions from these accounts may be the use of neurocomputational models, such as that developed by Dailey and colleagues (Dailey et al., 2010). Their model was trained in a Japanese or an American cultural context, and then tested with facial stimuli from both groups. The results of the model replicated the human in-group advantage for emotional facial expressions, lending support to an explanation emphasizing physical differences in the expressions of cultural groups. Recent evidence suggests that computer models may also be able to identify ethnic groups from speech segments (Hanani et al., 2013). Further computational models may add yet more to our understanding of how differences in emotional communication might arise in different cultural learning environments by incorporating possible motivational mechanisms.

### Conflict of interest statement

The author declares that the research was conducted in the absence of any commercial or financial relationships that could be construed as a potential conflict of interest.

## Appendix

**Table A1:** **Performance on the emotion recognition task in Experiment 1, with accuracy for Dutch, British, and Namibian stimuli shown for each emotion separately**.

**Origin**	**Emotion**			
	**Achievement**	**Amusement**	**Sensual pleasure**	**Relief**
Dutch	0.718 (0.225)	0.914 (0.137)	0.796 (0.187)	0.711 (0.252)
British	0.778 (0.242)	0.881 (0.162)	0.808 (0.152)	0.775 (0.195)
Namibian	0.316 (0.244)	0.655 (0.161)	0.451 (0.140)	0.446 (0.231)

Scores denote raw Hu scores, with standard deviations in brackets.
